# Atypical Presentation of Acute Appendicitis in a Young Adult With Congenital Adrenal Hyperplasia on Chronic Glucocorticoid Therapy

**DOI:** 10.7759/cureus.107303

**Published:** 2026-04-18

**Authors:** Carlos Diaz-Sepulveda, Luis A Santiago-Sulsona, Jose Nieves-Munoz, Guillermo Bolanos-Avila

**Affiliations:** 1 Surgery, Centro Médico Episcopal San Lucas, Ponce, PRI; 2 General Surgery Residency, Centro Médico Episcopal San Lucas, Ponce, PRI; 3 General Surgery, Centro Médico Episcopal San Lucas, Ponce, PRI

**Keywords:** acute appendicitis, alvarado score limitations, atypical appendicitis presentation, blunted inflammatory response, corticosteroid-induced masking of symptoms, cross-sectional imaging, diagnostic delay, glucocorticoid therapy, immunosuppression

## Abstract

We report the case of a 32-year-old man with congenital adrenal hyperplasia on chronic dexamethasone and fludrocortisone therapy who presented with nausea, diarrhea, and a single episode of vomiting, while denying abdominal pain, fever, anorexia, or right lower quadrant discomfort. Physical examination revealed a completely benign abdomen without tenderness, guarding, rebound, or peritoneal signs. Laboratory evaluation demonstrated hyponatremia and acute kidney injury without leukocytosis. Despite an Alvarado score of 1, CT imaging of the abdomen revealed acute appendicitis, which was later confirmed intraoperatively as a non-perforated, inflamed appendix. This case demonstrates an atypical presentation of acute appendicitis characterized by a benign abdominal examination and normal inflammatory markers in the setting of chronic glucocorticoid therapy. It highlights the importance of maintaining diagnostic vigilance and considering early imaging in steroid-dependent patients with persistent gastrointestinal symptoms, even in the absence of classic clinical findings.

## Introduction

Acute appendicitis is one of the most common surgical emergencies, with a lifetime risk of approximately 8-9% [1,2]. Its clinical progression has been classically described as visceral periumbilical, colicky pain that later localizes to the right lower quadrant as inflammation extends to the parietal peritoneum, producing localized tenderness, guarding, and rebound [1,2]. These findings form the basis of commonly used diagnostic tools, including the Alvarado score. This score is a widely used clinical scoring system designed to aid in the risk stratification of patients with suspected acute appendicitis by integrating symptoms, physical examination findings, and laboratory values. In patients without significant comorbidities, low scores are generally associated with a low probability of appendicitis and may support conservative management or observation, while higher scores prompt further diagnostic evaluation or surgical consultation. This approach has been shown to be useful in typical clinical settings, particularly when inflammatory markers and localized abdominal findings are present [3].

Delayed diagnosis of acute appendicitis is associated with a significantly increased risk of complications, including perforation, intra-abdominal abscess formation, generalized peritonitis, sepsis, and prolonged hospitalization [1,4]. Perforation rates rise substantially with increasing symptom duration, particularly beyond 36-48 hours, and are associated with higher morbidity, longer operative times, and increased postoperative complications [4]. Early diagnosis and timely surgical intervention are therefore critical to prevent disease progression and reduce adverse outcomes [1]. In patients with atypical or muted clinical presentations, failure to recognize appendicitis early may result in diagnostic delay and presentation at a more advanced and complicated stage.

The reliability of this diagnostic framework decreases in patients with suppressed inflammatory physiology. Chronic glucocorticoid therapy attenuates immune and inflammatory responses by reducing cytokine production, inhibiting prostaglandin synthesis, impairing leukocyte migration, and blunting febrile responses [5]. As a result, conditions that typically produce characteristic signs and symptoms may present atypically or with minimal findings. Altered presentations of appendicitis have been described in immunocompromised populations such as transplant recipients, oncology patients, and older adults [6-8].

Congenital adrenal hyperplasia (CAH), most commonly caused by 21-hydroxylase deficiency, requires lifelong glucocorticoid therapy to suppress excess adrenocorticotropic hormone and adrenal androgen production [9]. Patients with CAH lack the ability to mount an endogenous cortisol response to physiologic stress, placing them at risk for adrenal crisis during acute illness or surgery. Potent long-acting agents such as dexamethasone are frequently used and may exert sustained immunosuppressive effects [5,10,11]. Dexamethasone possesses approximately 25-30 times the glucocorticoid potency of hydrocortisone and has negligible mineralocorticoid activity, contributing to profound suppression of inflammatory and pain responses [5,11,(new1)]. While the endocrine and metabolic aspects of CAH are well established, the impact of chronic glucocorticoid therapy on the clinical presentation of acute intra-abdominal pathology has not been well characterized.

Diagnostic scoring systems may be particularly unreliable in this population. The Alvarado score depends on leukocytosis, fever, and localized tenderness [3], features that may be absent or blunted in patients receiving long-term steroids. A low score may therefore provide false reassurance. For this reason, diagnostic guidelines for immunocompromised patients emphasize a low threshold for imaging when the clinical picture is incomplete or discordant [12]. CT imaging provides objective diagnostic assessment and typically establishes the diagnosis of acute appendicitis through identification of dilated appendix, generally exceeding 6mm in diameter, accompanied by secondary inflammatory findings such as periappendiceal fat stranding, wall thickening, hyperenhancement, or the presence of an appendicolith [13].

This case report describes a completely painless presentation of acute appendicitis in a young adult with CAH receiving chronic glucocorticoid therapy. It highlights the limitations of physical examination and scoring systems in this setting and underscores the importance of early imaging when physiologic responses are suppressed.

## Case presentation

A 32-year-old man with CAH due to 21-hydroxylase deficiency, managed since childhood with dexamethasone 0.5 mg daily and fludrocortisone 0.1 mg daily, presented to the emergency department with one day of nausea, several episodes of loose stools, decreased oral intake, and a single episode of non-bloody, non-bilious vomiting. He denied abdominal pain, fever, chills, anorexia, dysuria, or right lower quadrant discomfort. He reported feeling well the previous day, had no recent travel or sick contacts, and had not started any new medications.

On arrival, his vital signs were within normal limits, and he appeared comfortable and in no distress. Abdominal examination revealed a soft, non-distended, and easily compressible abdomen without tenderness on light or deep palpation. There was no guarding, rebound, rigidity, or percussion tenderness. McBurney’s point tenderness, Rovsing’s sign, psoas sign, obturator sign, and heel-drop test were all negative. The patient consistently denied pain during repeated examinations performed by multiple clinicians.

**Figure 1:**
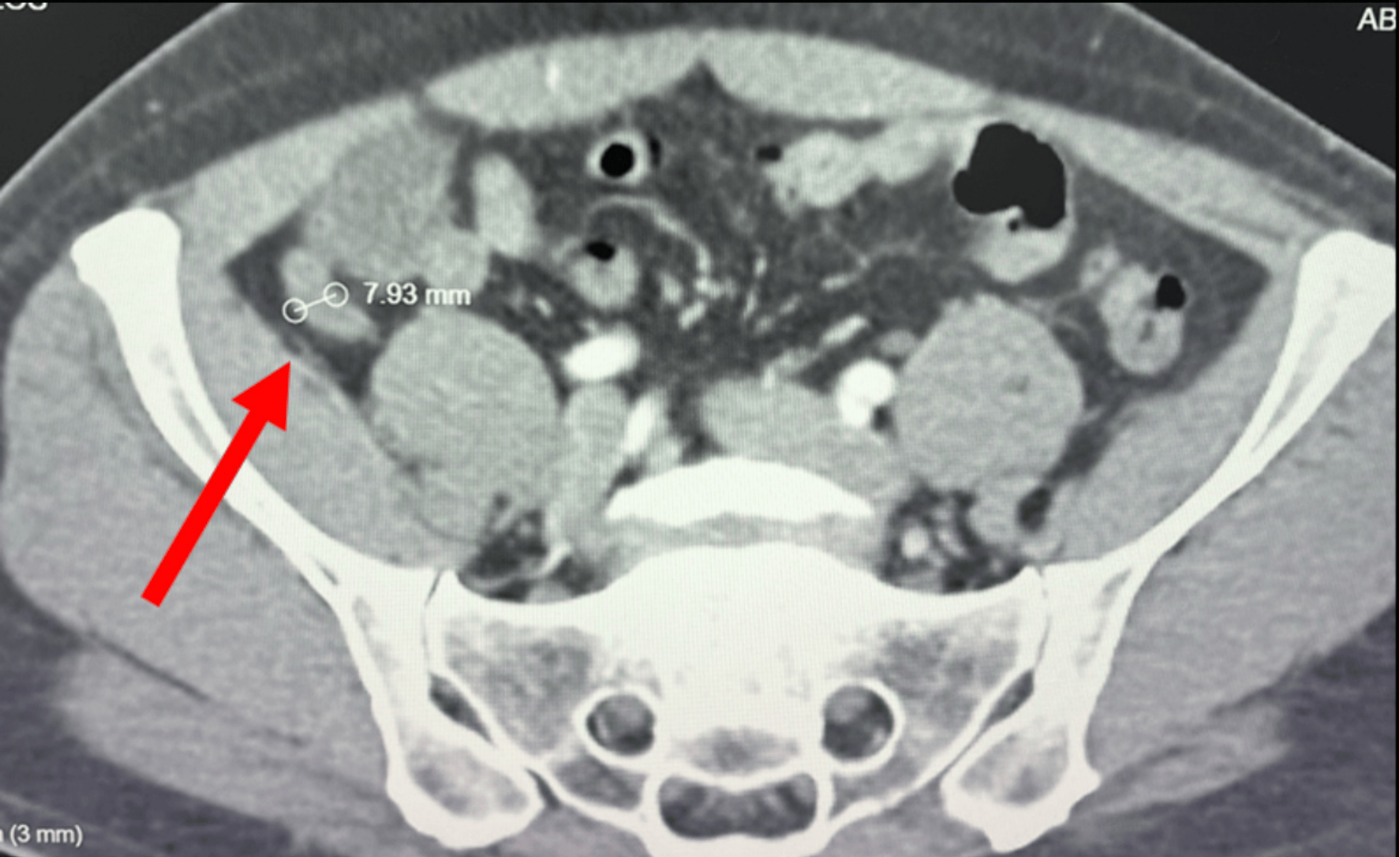
Contrast-enhanced CT of the abdomen demonstrating an enlarged appendix. Axial contrast-enhanced CT image of the abdomen demonstrating an enlarged appendix measuring approximately 7.9 mm in diameter (arrow), consistent with acute appendicitis.

**Figure 2:**
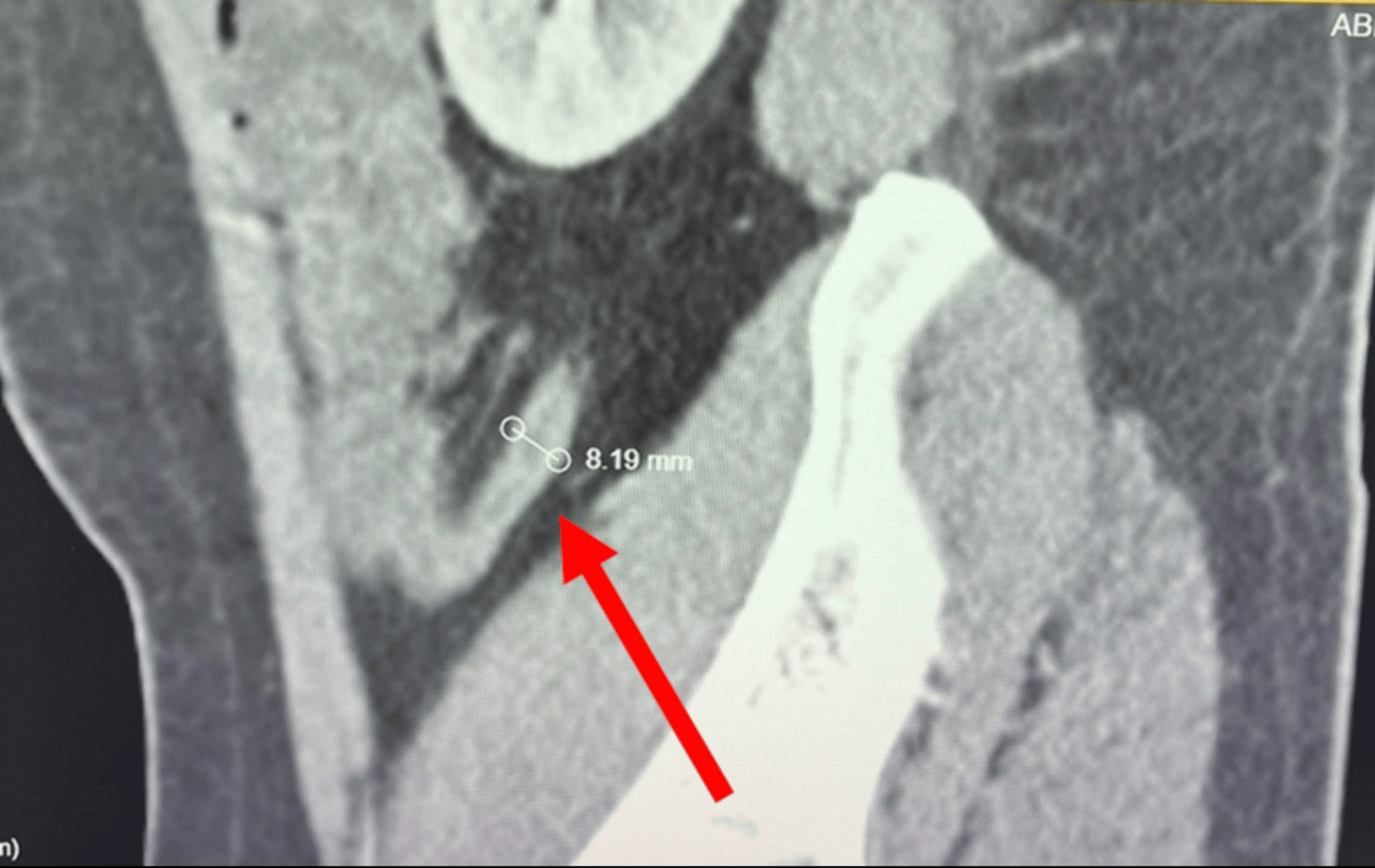
Contrast-enhanced CT of the abdomen demonstrating appendiceal enlargement. Sagittal contrast-enhanced CT image of the abdomen demonstrating an enlarged appendix measuring approximately 8.2 mm in diameter (arrow), consistent with acute appendicitis.

**Figure 3:**
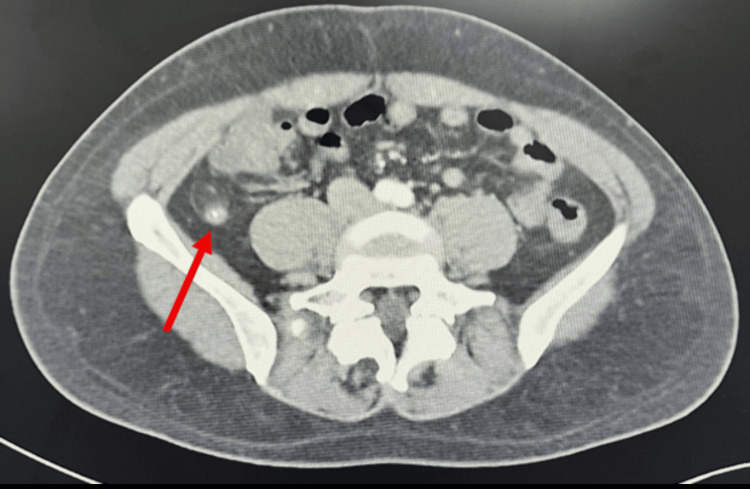
CT showing an appendicolith at the distal appendiceal tip. Axial contrast-enhanced CT image of the abdomen demonstrates a hyperdense appendicolith located at the distal tip of the appendix (arrow), with associated inflammatory changes consistent with acute appendicitis.

Laboratory evaluation demonstrated hyponatremia (126 mmol/L) and acute kidney injury (creatinine 2.0 mg/dL), which were initially interpreted as secondary to volume depletion from poor oral intake, diarrhea, and vomiting. Given the patient's history of CAH, early adrenal insufficiency was considered; however, the absence of hypotension, hypoglycemia, hyperkalemia, or hemodynamic instability favored dehydration as the primary etiology (Table 1). Despite the benign abdominal examination, the history of gastrointestinal symptoms and metabolic abnormalities prompted further evaluation. CT of the abdomen/pelvis revealed an enlarged, inflamed appendix with periappendiceal fat stranding and the presence of appendicolith, consistent with acute appendicitis (Figures 1-3). The patient’s Alvarado score was calculated as 1 point, attributed solely to nausea and vomiting (Table 2).

**Table 1:** Laboratory investigations on initial presentation. Summary of laboratory investigations obtained on initial presentation. Reference ranges correspond to institutional laboratory standards.

Laboratory test	Patient value	Reference range	Units
White blood cell count (WBC)	7.44	4.0–11.0	×10³/µL
Hemoglobin	12.6	12.0–16.0	g/dL
Hematocrit	35.3	36–48	%
Platelet count	127	150–400	×10³/µL
Sodium	126	135–145	mmol/L
Potassium	4.3	3.5–5.0	mmol/L
Chloride	90	98–106	mmol/L
Bicarbonate	22.3	22–29	mmol/L
Blood urea nitrogen	35	7–20	mg/dL
Creatinine	2	0.6–1.3	mg/dL
Glucose	91	70–100	mg/dL

**Table 2:** Alvarado score and interpretation. The Alvarado score is a clinical scoring system used to aid in the risk stratification of patients with suspected acute appendicitis by integrating symptoms, physical examination findings, and laboratory parameters. Scores range from 0 to 10, with higher scores indicating a greater likelihood of appendicitis. Scores of 1-4 suggest low risk, 5-6 intermediate risk, and 7-10 high risk, guiding decisions regarding observation, imaging, or urgent surgical consultation [3].

Alvarado score for acute appendicitis	
+2 points - Right lower quadrant tenderness	
+1 point - Elevated temperature (>37.3°C or 99.1°F)	
+1 point - Rebound tenderness	
+1 point - Migration of pain to the right lower quadrant	
+1 point - Anorexia	
+1 point - Nausea or vomiting	
+2 point - Leukocytosis > 10,000	
+1 point - Leukocyte left shift	
1-4 (Low risk): Appendicitis is very unlikely. Alternative diagnoses should be considered, and outpatient management may be appropriate.	
5-6 (Intermediate risk): Appendicitis is possible. Active observation, further imaging (e.g., ultrasound or CT), or serial clinical exams are recommended.	
7-10 (High risk): Appendicitis is highly likely. Urgent surgical consultation is required, as a score of is considered diagnostic of acute appendicitis.	

Despite the low score, surgical consultation recommended operative management, given CT evidence of appendicitis. He underwent laparoscopic appendectomy and intraoperative findings demonstrated an erythematous and inflamed appendix without evidence of perforation, abscess, gangrene, or free intraperitoneal fluid. Histopathological examination confirmed acute appendicitis with transmural neutrophilic infiltration. The patient did not receive perioperative glucocorticoid supplementation and did not receive intravenous hydrocortisone or endocrine consultation. Due to same-day discharge, no inpatient steroid dosing was administered. He remained hemodynamically stable, without clinical or lab evidence of adrenal insufficiency. His postoperative course was uncomplicated, and he was discharged home the same day.

## Discussion

This case illustrates how chronic glucocorticoid therapy can significantly alter the clinical presentation of acute appendicitis and complicate timely diagnosis. Despite clear radiographic, intraoperative, and histopathologic evidence of appendiceal inflammation, this patient had no abdominal pain, peritoneal signs, fever, or leukocytosis. Rather than indicating the absence of disease, these findings reflect suppression of the normal inflammatory response, creating a deceptively benign clinical picture.

The diagnosis of appendicitis traditionally relies on a predictable progression of symptoms and signs, including migration of pain to the right lower quadrant, localized tenderness, and systemic inflammatory markers. Clinical scoring systems such as the Alvarado score are built on this framework and perform reasonably well in immunocompetent patients. However, these tools depend on physiologic responses that may be attenuated by long-term glucocorticoid exposure. Glucocorticoids reduce cytokine signaling, inhibit leukocyte migration, and suppress febrile and pain responses, which can obscure classic manifestations of acute intra-abdominal inflammation [5,10,11].

Atypical presentations of appendicitis have been described in immunocompromised populations and in elderly patients, in whom delayed diagnosis and increased complication rates are well documented [6-8]. In contrast, there is limited literature specifically examining the impact of chronic glucocorticoid therapy as an independent factor in the clinical presentation of acute appendicitis in patients with CAH. There are reported cases of patients receiving long-term steroids for other conditions, such as systemic lupus erythematosus and rheumatoid arthritis, who present with similar blunted presentations in acute abdominal pathologies, but are often grouped within broader immunocompromised cohorts [new 2]. This limits the ability to isolate steroid therapy as a distinct contributor to diagnostic masking.

The calculated Alvarado score of 1 in this patient demonstrates the limitations of applying standard diagnostic scoring systems without consideration of underlying physiology. In most clinical settings, such a low score would strongly argue against appendicitis and favor observation or supportive care. In patients receiving chronic steroids, the absence of leukocytosis, fever, or localized tenderness may reflect pharmacologic immune suppression rather than low disease probability. When interpreted in isolation, scoring systems in this population may therefore be misleading and contribute to diagnostic delay.

In this case, CT imaging was essential in establishing the diagnosis. Imaging revealed appendiceal inflammation despite a benign abdominal examination and low clinical score, allowing surgical intervention before disease progression. Current guidelines addressing the evaluation of immunocompromised patients emphasize a low threshold for cross-sectional imaging when symptoms are unexplained or out of proportion to physical findings, recognizing that traditional markers of inflammation may be unreliable [12,13]. This principle is directly applicable to patients on chronic glucocorticoid therapy.

This case adds to the limited evidence suggesting that long-term steroid use in CAH patients can mask acute intra-abdominal pathology and reduce the diagnostic utility of traditional clinical frameworks. Recognition of this altered physiologic response is critical. Clinicians should maintain heightened suspicion and consider early imaging when evaluating steroid-dependent CAH patients with gastrointestinal symptoms, even when classic features of appendicitis are absent.

## Conclusions

Acute appendicitis may present without pain, tenderness, or laboratory abnormalities in patients receiving long-term glucocorticoid therapy. In individuals with CAH, suppressed inflammatory responses can obscure classic clinical features and render diagnostic scoring systems unreliable. Clinicians should maintain a high index of suspicion and consider early imaging when evaluating steroid-dependent patients with persistent gastrointestinal symptoms, even in the absence of typical findings, to prevent diagnostic delay and associated complications.
